# The Influence of Auditory Information on Visual Size Adaptation

**DOI:** 10.3389/fnins.2017.00594

**Published:** 2017-10-24

**Authors:** Alessia Tonelli, Luigi F. Cuturi, Monica Gori

**Affiliations:** ^1^Unit for Visually Impaired People, Science and Technology for Children and Adults, Istituto Italiano di Tecnologia, Genoa, Italy; ^2^Robotics, Brain and Cognitive Sciences Department, Istituto Italiano di Tecnologia, Genoa, Italy

**Keywords:** size perception, multisensory integration, aftereffects, audiovisual integration, auditory cue, visual perception

## Abstract

Size perception can be influenced by several visual cues, such as spatial (e.g., depth or vergence) and temporal contextual cues (e.g., adaptation to steady visual stimulation). Nevertheless, perception is generally multisensory and other sensory modalities, such as auditory, can contribute to the functional estimation of the size of objects. In this study, we investigate whether auditory stimuli at different sound pitches can influence visual size perception after visual adaptation. To this aim, we used an adaptation paradigm (Pooresmaeili et al., 2013) in three experimental conditions: visual-only, visual-sound at 100 Hz and visual-sound at 9,000 Hz. We asked participants to judge the size of a test stimulus in a size discrimination task. First, we obtained a baseline for all conditions. In the visual-sound conditions, the auditory stimulus was concurrent to the test stimulus. Secondly, we repeated the task by presenting an adapter (twice as big as the reference stimulus) before the test stimulus. We replicated the size aftereffect in the visual-only condition: the test stimulus was perceived smaller than its physical size. The new finding is that we found the auditory stimuli have an effect on the perceived size of the test stimulus after visual adaptation: low frequency sound decreased the effect of visual adaptation, making the stimulus perceived bigger compared to the visual-only condition, and contrarily, the high frequency sound had the opposite effect, making the test size perceived even smaller.

## Introduction

Humans rely on visual information to create a representation of the environment. Perception of size is an important feature to build this representation, but, like other features, it can be subjected to an erroneous perception due to several multi-sensory information. Size perception depends on several factors: spatial context (Leibowitz et al., 1969; Aglioti et al., 1995; Haffenden et al., 2001; Konkle and Oliva, 2012), vergence (Bradshaw et al., 1996; Arnold et al., 2008) and visual adaptation (Blakemore and Sutton, 1969; Pooresmaeili et al., 2013), leading to a misperception of physical size. In this work, we will investigate the influence of adaptation on size perception.

Adaptation is a behavioral technique used to investigate the flexibility of the neural responses to a specific sensory stimulation (for a review see Thompson and Burr, 2009). For example, a prolonged presentation of a stimulus (adapter) with constant size can affect the size of a subsequent target presented in the same portion of space. If the adapter is bigger than the target, the latter will be perceived smaller than its physical size and vice versa, if the adaptation is performed with a small stimulus the target will be perceived bigger. This process is called size adaptation or size aftereffect (Pooresmaeili et al., 2013).

Visual perception is not just influenced by visual information, but can also be greatly altered by other sensory modalities, even at early stages of processing (for a review see Shams and Kim, 2010). Auditory or tactile information has an impact on several visual tasks, such as the visual detection task (Frassinetti et al., 2002; Bolognini et al., 2005), contrast sensitivity (Tanaka et al., 2005; Lippert et al., 2007), and motion perception (Meyer and Wuerger, 2001; Hidaka et al., 2009; Teramoto et al., 2010). Another example of such interaction across modalities is given by the bouncing illusion (Sekuler et al., 1997). When two identical objects move along the diagonal of a 2-dimensional display, the objects can be seen either bouncing against each other or crossing along a stream. However, when the two objects coincide and a brief sound is simultaneously presented, the visual perception of motion is biased toward the bouncing motion. The reason behind this perceptual switch is not clear, but this phenomenon can also occur with subliminal sounds, suggesting a perceptual level of processing (Dufour et al., 2008).

Adaptation can also be transferred between different sensory modalities, leading to cross-modal aftereffects by using a visual/tactile motion adaptation (Konkle et al., 2009), vestibular/proprioceptive motion adaptation (Cuturi and Macneilage, 2014) and visual/auditory depth motion adaptation (Kitagawa and Ichihara, 2002).

Similarly, audition can influence perception of size, showing cross-modal synesthetic association. Evidence of such interactions between sound frequencies and visual size show that irrelevant sounds could influence the speed with which participants judged the size of visual stimuli (Gallace and Spence, 2006; Parise and Spence, 2009). Takeshima and Gyoba (2013) tested which aspects between sound intensity, temporal windows of audio-visual interaction and eccentricity of the visual stimulus could been involved in the influence of auditory stimuli on visual perception of size. They found that high-intensity auditory stimuli increased the perceived size of an object and that this bias is limited to a narrow temporal window (−100 to +100 ms), and that this effect was greater in the peripheral visual field.

No studies to date have investigated whether similar associations are also present for the integration between vision and audition with regard to size aftereffect. Here we investigated this point by testing the influence of auditory cues on visual size aftereffect.

We used a similar paradigm of size adaptation as that used by Pooresmaeili et al. (2013). Participants had to perform a size discrimination task in three experimental conditions: visual-only, visual-sound at 100 Hz and visual-sound at 9,000 Hz. The participants had to judge which of the two stimuli was bigger. Visual stimuli were presented in the periphery of the visual field. In the main experiment, the auditory stimulus was concurrent to the test stimulus. For all conditions, we repeated the task, presenting an adapter (twice as big as the reference stimulus) before the test stimulus. In this way we had a congruent and an incongruent condition, i.e., the high pitch and adaptation would facilitate or increase the effect, that is, the test stimulus would be perceived as smaller, whereas the low pitch would decrease the effect induced by adaptation. We run also a control experiment with a reduced sample of participants in which there was a temporal delay between test and auditory stimuli. We wanted to test whether the effect was modulated by stimulus onset asynchrony.

Before the size discrimination task, the participants participated in a “listen and draw” task, in which they listened to sounds of different frequencies and had to draw on a screen a circle that matched the size expressed by the sound presented.

We hypothesized that in the “listen and draw” task, the participants would naturally match high frequency to small size and low frequency to big size. In the size discrimination task, we expected an increase in the aftereffect in the high pitch condition and a decrease in the aftereffect in the low pitch condition compared to the visual-only condition. Our results confirm our hypothesis: the low frequency sound decreased the effect of visual adaptation while the high frequency sound had the opposite effect.

## Materials and methods

### Participants

Sixteen healthy adults (eight male; average age of 25, *SD* = 3) with normal or corrected to normal vision were recruited to participate in our experiment. All participants gave written informed consent before starting the test. The study was approved by the ethics committee of the local health service (Comitato Etico, ASL 3, Genova).

### Apparatus and stimuli

For the listen and draw task, the sounds were generated by means of Audacity software (http://audacity.sourceforge.net/). We used five sounds with different pitches: 100, 500, 1,000, 5,000, and 9,000 Hz. The duration of the auditory stimulus was 500 ms. Visual stimuli were generated in MATLAB, using the Psychophysics Toolbox version extensions. The monitor on which the participants had to draw was a 19-inch LCD color monitor (resolution 1,280 × 1,024).

For the size discrimination task, stimuli were displayed on a Barco monitor (resolution of 1,280 × 1,024 corresponding to 41° × 31.2° from subject viewing distance of 57 cm with a refresh rate of 100 Hz).

In the size discrimination task, visual stimuli were Craik-O'Brien-Cornsweet stimuli. The stimuli were constructed by high-pass Gaussian filters (with a 50% cutoff at spatial frequency of 0.5 cycles/°), with the advantage of eliciting localized activation of the visual cortex, limited to their edges (Perna et al., 2005; Boyaci et al., 2007; Pooresmaeili et al., 2013). All visual stimuli were displayed with an eccentricity of 10°. The test stimulus was always presented on the left of the screen, whereas the reference stimulus was presented with the same eccentricity on the right side. The adapter stimulus was always centered at the same eccentricity as the test stimulus. The size of the adapter and the reference stimuli were keep constant, at 10° and 5° respectively. The size of the test stimulus was determined by QUEST (Watson and Pelli, 1983), an adaptive algorithm which, based on the current estimation of the participant, estimates the best stimulus value to be presented in the subsequent trial. The range of variation of the test stimulus was between 0.5 to 1.5 times the size of reference stimulus. Therefore, the physical size of the test stimulus was always smaller than the adapter. The test and the reference stimuli were presented for 500 ms, while the adapter lasted 10 s with a top-up of 6 s. For the auditory conditions, we used two of the sounds used in the previous task: the lowest pitch (100 Hz) and the highest pitch (9,000 Hz).

In all tasks, sounds were heard through noise canceling headphones (Bose® QuietComfort 25 Acoustic Noise Cancelling Headphones).

### Procedure

**Figure 1:**
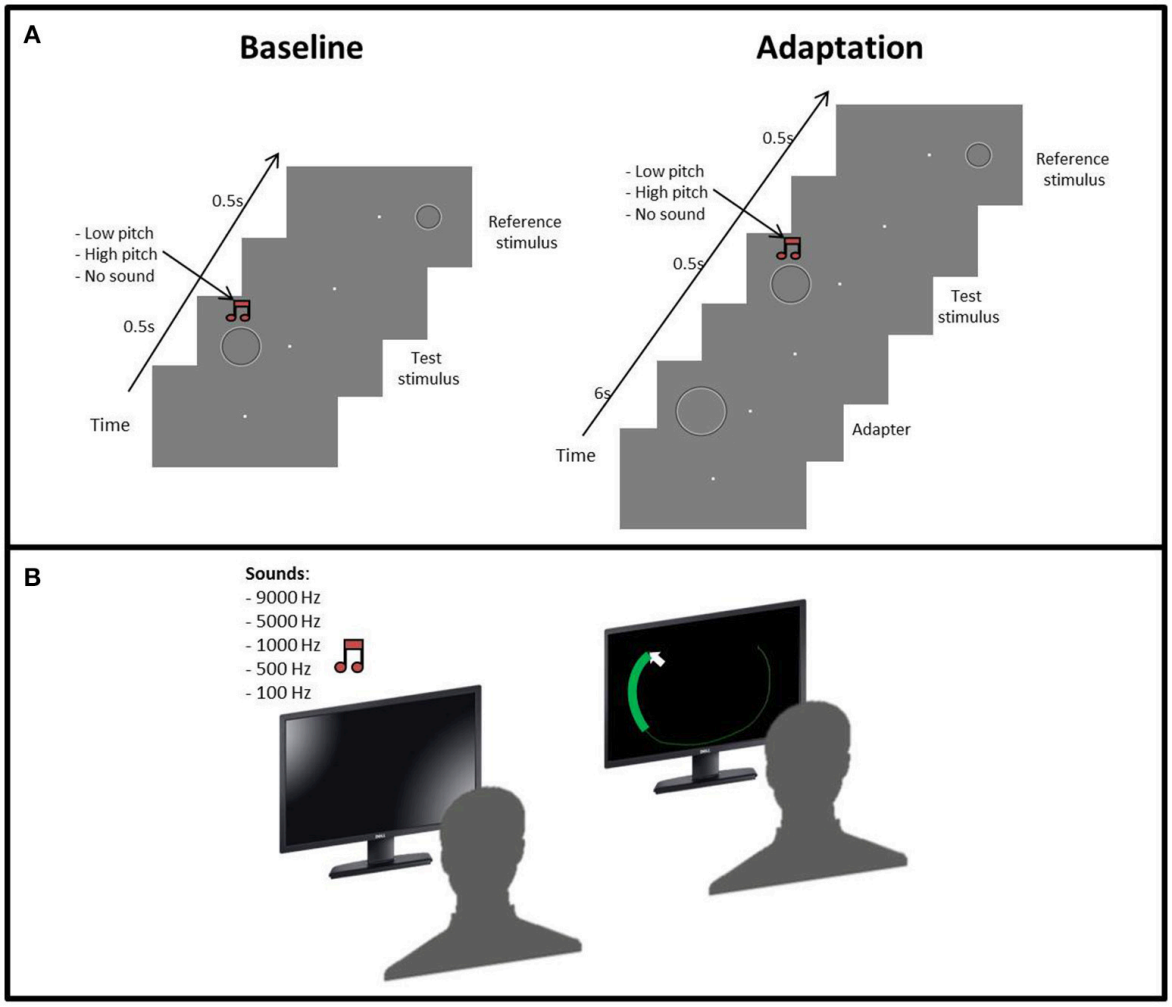
**(A)** Procedure of the size adaptation paradigm: the first phase started with a gray screen and a fixation point in the middle of the screen, then the test stimulus and the reference stimulus were subsequently presented. Concurrent with the test stimulus, an acoustic sound (low or high frequency) could also be presented with the same duration of the test stimulus. In the second phase, the adapter was presented before the test stimulus. **(B)** Set up for the “listen and draw” task. First, the participant hears on of the sounds and then using the mouse has to draw a circle as big as the sound heard.

First, each participant performed “the listen and draw” task. The participant sat in front of a screen and was asked to draw a circle as big as the sound he/she heard by pressing the left button of the mouse (Figure 1B). Participants performed 25 trials, 5 repetitions per pitch level.

The size discrimination experiment was divided into two phases. In the first one the subject was presented with three blocks of trials, one for each condition, in a pseudo-randomized order. In the visual condition, we evaluated the perceptual bias of each participant in discriminating the size test and reference stimuli (Figure 1A). In the sound conditions, we simultaneously presented the low (100 Hz) or high (9,000 Hz) pitch sounds with the test stimulus. Each condition consisted of 100 trials.

The second phase was identical to the first, except that before the test stimulus we presented the adapter stimulus. Also, in this case the sequence of the blocks was pseudo-randomized. Each condition consisted of 75 trials.

In all phases, the participants had to fix the fixation point on the center of the screen and judge whether the test stimulus was bigger or smaller than the reference by pressing a key on the keyboard (L = bigger, S = smaller).

A reduced sample of participants was tested again to repeat the size discrimination task. In this case in the auditory conditions the test and the auditory stimuli were presented with an asynchrony of 80 ms (as in Jaekl et al., 2012), with the visual stimulus leading.

## Results

About the size discrimination task, first we ran an analysis to all conditions to check for the presence of outliers in the sample. One participant had an adaptation effect higher than two standard deviation from the mean in the incongruent condition (*M* = 10.2%, *SD* = 3.4%; participant = 18%), so was excluded from further the analysis.

**Figure 2:**
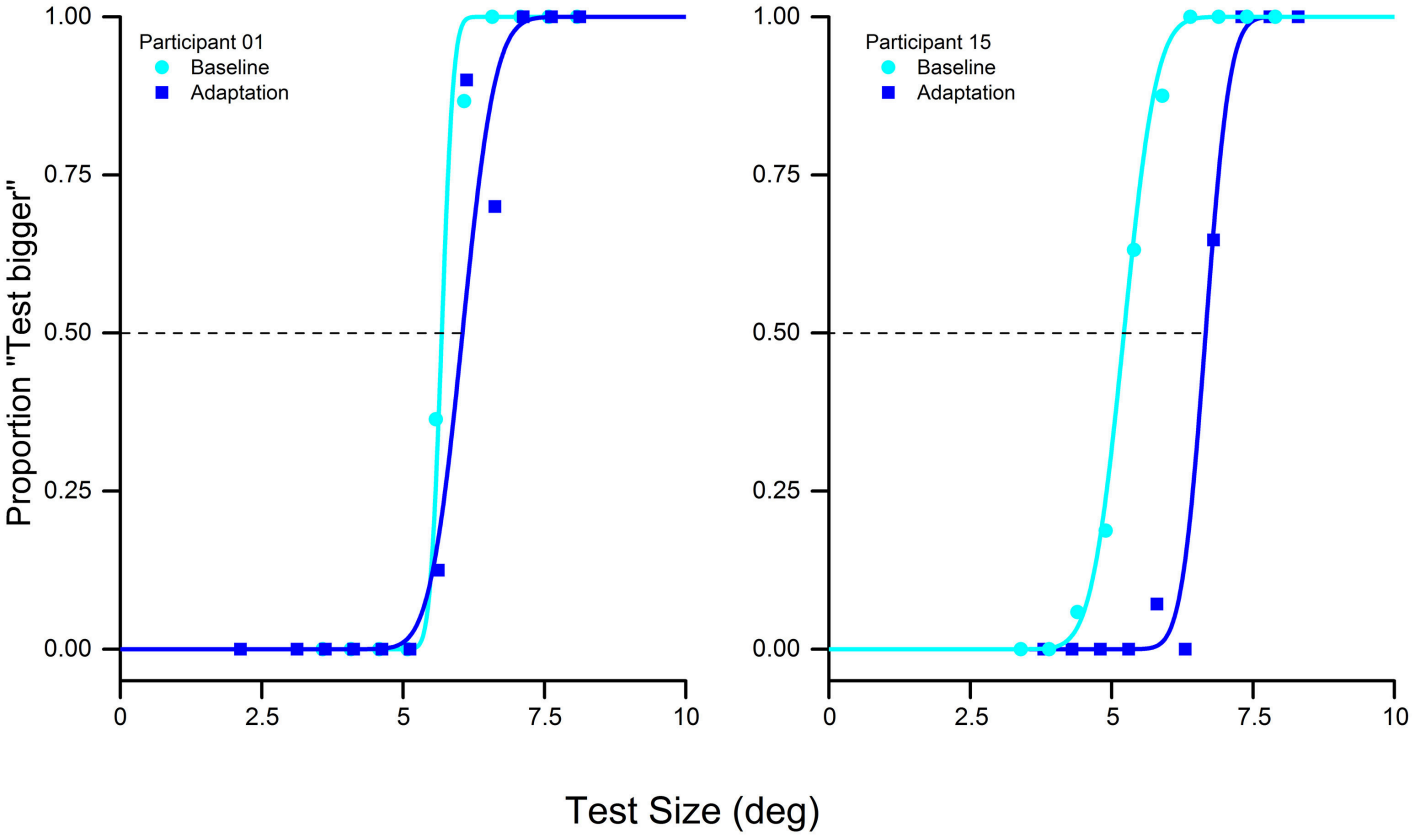
Psychometric discrimination function of the baseline (cyan) and adaptation (blue) in the visual-only condition of two typical subjects: adaptation to a 10° stimulus with a reference of 5° produces a rightward shift of the psychometric curve, indicating that the adaptation reduces the perceived size of the test stimulus.

We replicated the behavioral results obtain by Pooresmaeili et al. (2013). The presentation of the adapter stimulus influenced the perceived size of the subsequent test stimulus. The adapter, always bigger than the test, produced a shift of the psychometric function compared to the baseline, thus showing a decrease in the perceived size of the test stimulus (Figure 2). This result was supported by the statistical analysis that confirmed a significant difference between the point of subject equality (PSE) of the baseline and the adaptation in the visual condition [two tailed pair-sample *t*-test, *t*_(15)_ = −9.1, *p* < 0.0001].

**Figure 3:**
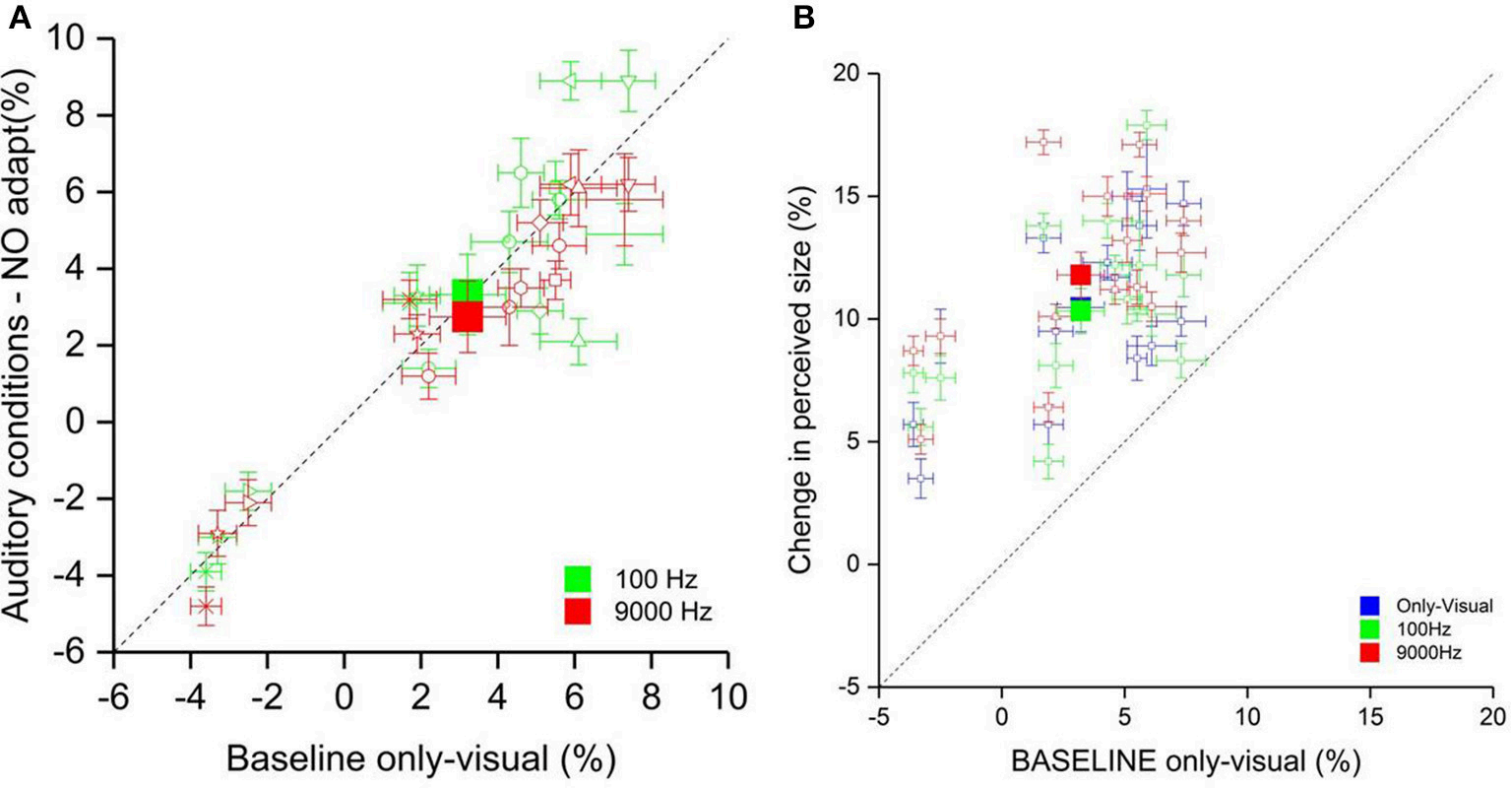
**(A)** The scatter plot shows the percentage of the perceived size of the visual stimulus in the auditory conditions (on the ordinate) and the only-visual condition (on the abscissa). The filled squares represent the average of the low pitch condition (green) and high pitch condition (red). The empty symbols are the data of single participants. **(B)** The scatter plot represents the percentage of the perceived size of the visual stimulus in all conditions after the visual adaptation (on the ordinate) and the only-visual condition (on the abscissa) without adaptation. The filled squares represent the average of the low pitch (green), high pitch (red) and only-visual (blue) conditions. The empty symbols are the data of single participants.

First, we analyzed only the effect of the low and high pitch sound on the perceived size of the visual stimuli (phase one). We ran a one-way ANOVA with factor Condition (visual-only, high and low pitch). The results are showed in Figure 3A did not have a significant outcome [*F*_(3, 15)_ = 1.21, *p* = 0.31].

Subsequently, we calculated the effect of adaptation for all three conditions (visual-only, low and high pitch). The only-visual condition acquired in the first phase was considered as baseline. We defined as Δ of the effect the difference of the PSEs of each adaptations and the baseline. The results are plotted in Figure 3B. The Δ of the effect for the sound conditions showed an opposite tendency: the low pitch sound presented simultaneously to the visual test made the test stimulus be perceived bigger compared to in the visual-only condition, whereas the high frequency sound made it be perceived even smaller. A one-way ANOVA with factor Condition (visual-only, high pitch, and low pitch) showed a main effect [*F*_(3, 15)_ = 5, *p* < 0.02]. *Post-hoc* analysis (two tailed pair-sample *t*-test) Bonferroni corrected for multiple comparison reflected a significant difference between the two sound conditions [*t*_(15)_ = −3.61, *p* < 0.01] and between the visual-only and the high pitch conditions [*t*_(15)_ = 2.96, *p* < 0.05]. Moreover, we wanted to test whether there is a correlation between the effect of adaptation and the pitch, that could explain the variability between participants. We run a Pearson correlation analysis between the Δ of the effect in the only-visual condition and the sound conditions. We have a positive correlation of 0.79 (*p* < 0.0001) between the only-visual and low pitch conditions and of 0.82 (*p* < 0.0001) between the only-visual and high pitch conditions, i.e., more a participant is affected by contextual visual information more this effect will increase by adding congruent auditory information.

**Figure 4:**
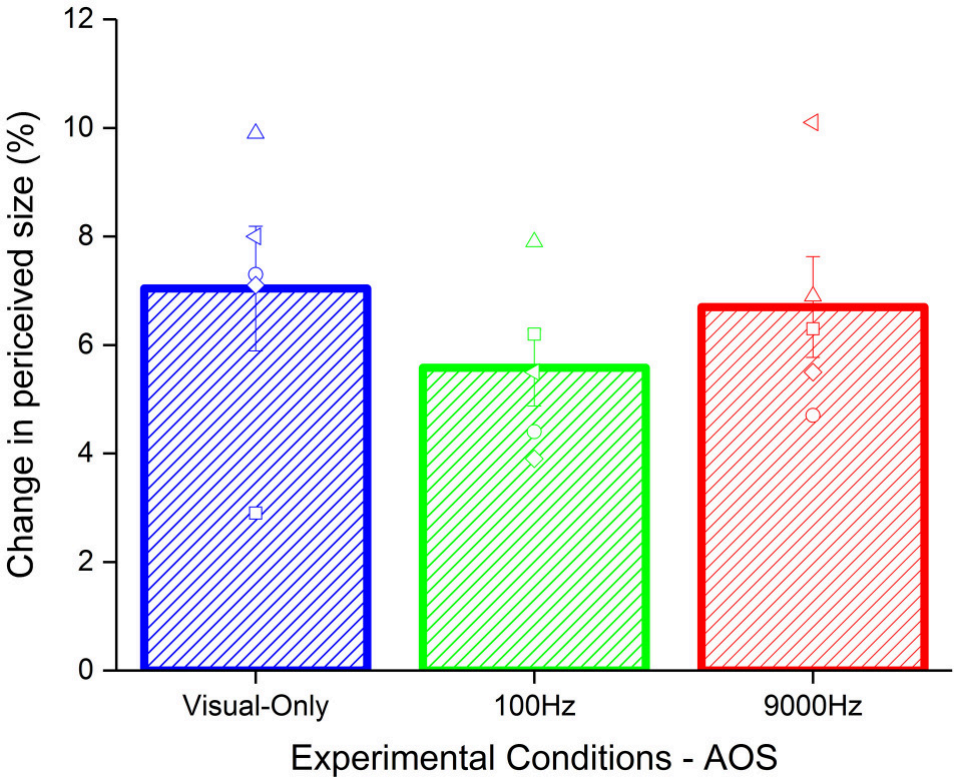
Average of the aftereffect for each condition with temporal asynchrony: visual-only (blue), low pitch (green), and high pitch (red) frequencies frequency. The empty symbols are the data of single participants.

To test whether the effect found was due to the synchrony between acoustic and visual stimuli, we analyzed the results of a reduced sample (5 participants) where we introduced an asynchrony of 80 ms between the test and the acoustic stimuli. Given the small size of the sample we used a no parametric test, i.e., Kruskal–Wallis test. As for the synchronous condition, we calculated the Δ of the effect by subtracting the baseline to the PSEs of the adaptation for each condition (Figure 4). The results did not highlight a significant difference between the conditions (χ^2^ = 1.94, *p* = 0.37).

For the “listen and draw” test, we calculated the area drawn on the screen by converting the area from pixels to cm^2^. We ran a Lilliefors (Kolmogorov–Smirnov) test to check the normality of the sample for the “listen and draw” test. Results showed that data were not normally distributed for the area drawn.

**Figure 5:**
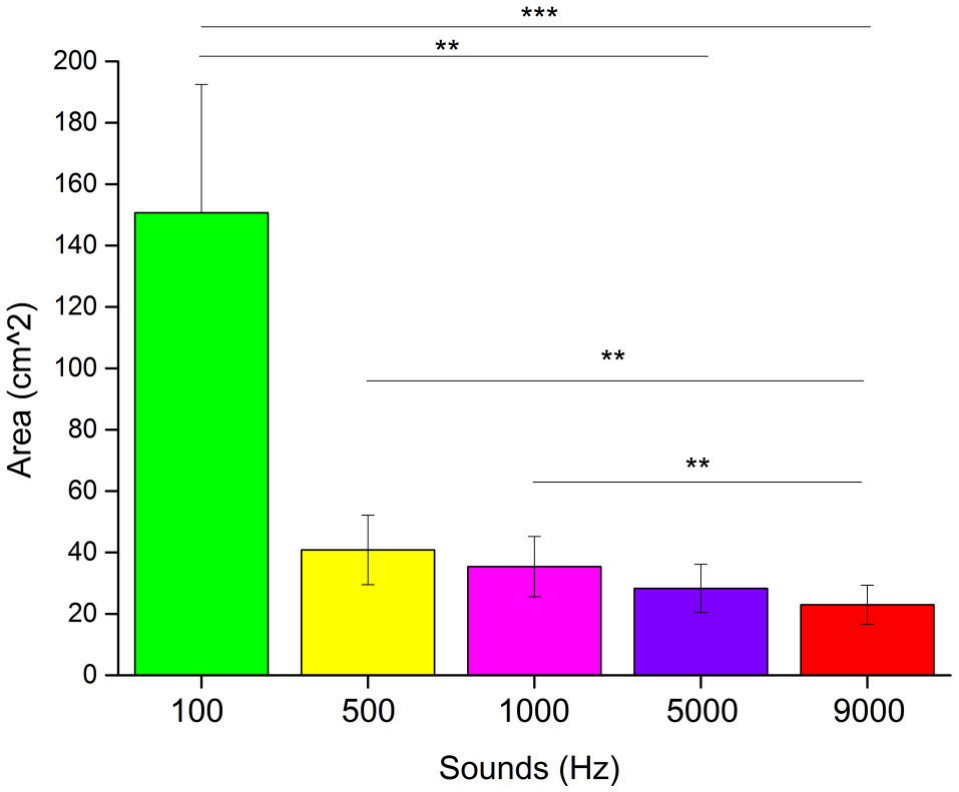
Results of the “listen and draw” task. Each bar represents the average area in cm^2^ drawn for each frequency of the sound heard. ^***^Significant difference with *p* < 0.001. ^**^Significant difference with *p* < 0.01.

We used non-parametric statistical analysis. To see if there was a difference in the areas drawn associated to each pitch, we performed a Kruskal–Wallis test analysis. Results (Figure 5) showed a main effect for the type of pitch heard (χ^2^ = 26.14, p < 0.0001). A *post-hoc* Bonferroni correction for multiple comparisons revealed a significant difference between the areas of 100 and 9,000 Hz (*p* < 0.001), 100 and 5,000 Hz (*p* < 0.01), 500 and 9,000 Hz (*p* < 0.01) and 1,000 and 9,000 Hz (*p* < 0.01).

## Discussion

Several studies have shown cross-modal associations between sound frequencies and visual features of objects, such as spatial positions, brightness, size and sharpness (Carello et al., 1998; Gallace and Spence, 2006; Parise and Spence, 2009; for a review Spence, 2011; Bien et al., 2012; Jaekl et al., 2012; Takeshima and Gyoba, 2013). Here we showed that a similar association can also be obtained by using cross-modal stimulations: namely, an audio pitch cue can be used to modulate visual size adaptation.

The main hypothesis of this study was to see whether sound frequencies can influence size aftereffect. To test our hypothesis, we used the size adaptation paradigm of Pooresmaeili et al. (2013) to create a size aftereffect, in which, after adaptation, the stimulus test was perceived differently from its physical size. In our study, we presented a sound (low or high frequency pure tone) at the same time as the test stimulus to evaluate whether the effect of the adaptation was modified or not. Pooresmaeili et al. (2013) tested visual adaptation for both small and big size, i.e., in the first case the adapter was smaller than the reference, in the second case the adapter was bigger. Since they found a greater effect for the condition in which the adapter was bigger than the reference stimulus, we decided to focus on this condition in our study.

As we expected, the results confirmed our hypothesis. In the congruent condition, i.e., high pitch sound associated with adaptation to big size, the test stimulus was perceived even smaller compared to the visual-only condition. This might happen because both high pitch sound and adapter have the same effect on the test stimulus, i.e., to be perceived as smaller than the reference. The opposite effect was found for the incongruent condition, where we found a decrease of the aftereffect associated to the low pitch sound, due to opposite effects, i.e., the low pitch sound might increase the perceived size of the visual test, while adaptation to a bigger stimulus makes the test stimulus be perceived smaller, thus showing a reduction of the aftereffect.

Several studies have shown how size perception can be influenced by the pitch of sounds (Mondloch and Maurer, 2004; Gallace and Spence, 2006; Parise and Spence, 2009; Evans and Treisman, 2011; Bien et al., 2012) or loudness (Jaekl et al., 2012; Takeshima and Gyoba, 2013), i.e., low pitch (or loudness) sounds are associated with big objects, while high pitch (or loudness) sounds with small ones. The novelty of our work consists of showing that pitch has an effect not only on size perception but also on the perceived size after visual adaptation. These results contribute to corroborating how human primary visual cortex activity is modulated by sound as a result of auditory-visual integration. It has been shown that non-visual sensory information can modulate visual perception in a different domain at the behavioral level: in the last decade several pieces of evidence have shown how cross-modal stimulation can affect the activity of the visual cortex (Lewis et al., 2000; Arden et al., 2003; Scheef et al., 2009; Murray et al., 2015). For example Molholm et al. (2002) found an ERP response to audiovisual stimuli at parieto-occipital/occipital position. Moreover, there are findings suggesting that the modulation of the visual cortex by sound occurs at the early stages of information processing, directly involving the primary visual cortex (Shams et al., 2000, 2001; Noesselt et al., 2007; Feng et al., 2014). In this study we have deliberately used an experimental paradigm that we know acts directly on V1. Aftereffect, *per-se*, is a behavioral technique used to investigate adaptability of the neural mechanism tuned (Frisby, 1980), in this specific case, to size perception. Furthermore, Pooresmaeili et al. (2013) suggested a primary, not auxiliary, role of the striate cortex in size adaptation based on a response gain model on local excitation and inhibition within area V1. Moreover, the adaptation paradigm did not use perspective or vergence cues to create size illusion, because the distance and the depth from the participant were kept constant. Therefore, the apparent size should not be attributable to simultaneous spatial contextual effects. Looking to single participants data, it is present a variability in effect produced by the association between adaptation and sounds. We found that there is a positive correlation between the Δ effect of only-visual adaptation and the Δ of auditory condition, meaning that the subjects that are more affected by only-visual adaptation are the ones that are more influenced also in the auditory conditions.

Given the lack of spatial congruency between the test and the auditory stimuli, i.e., the test stimulus is always presented on the left, instead the sound is listen with both ears, the temporal synchronization appear to be the main factor that bounce together these two stimuli is. We wanted to highlight this point because it suggests that temporal coincidence plays a key role to find the effect. To test this hypothesis, we ran a control experiment with a reduced part of the initial sample, where we put a temporal delay between the presentation of the test and sounds stimuli. We found no significant difference between the Δ effect of the auditory (low and high pitch) and the Δ effect of the only-visual conditions, suggesting that the effects of auditory pitch vanish after its presentation. If this was not the case, we would have found a greater effect as in Jaekl et al. (2012), where the authors found an increase of the perceived size of a circle when the sound followed the visual stimulus. So it seems that temporal synchronization is needed when the perception has already been altered by visual adaptation.

Another evidence of the cross-modal association between sound frequencies and size perception, is given by the “listen and draw” task. In this task we left the participants free to draw a circle as big as the sound they heard, in order to make the association as natural and ecological as possible, unlike for example discrimination task, in which the person is forced to choose between two or more stimuli. Taking into account the lowest frequency (100 Hz), it is clear how this frequency is associated with an extremely large circle, generating a gap with the others sounds. On the other hand, the same significant strong effect was not found with the highest frequency (9,000 Hz); although the area drawn was smaller, the gap was not as consistent as for the 100 Hz sound, in comparison with the other frequencies used. A possible explanation of this discrepancy can be given by the curves of Fletcher and Munson (1933). According to this approach, the perceived intensity of a sound can remain unaltered if frequency and sound pressure vary following these curves. For example, 1,000 Hz at 40 dB will be perceived with the same intensity as 100 Hz at 54 dB. It should be highlighted that the steepness of the curve decreases with increasing frequency. In our experiment, we kept sound pressure constant, therefore by varying the sound's frequency, the perceived intensity also changed. Given the Fletcher and Munson curves steepness, the difference in intensity is higher for low frequencies than high frequencies. This might explain why we found a bigger gap between the area drawn associated with 100 Hz compared to the others, where the size of the area decreases steadily as the frequency increases.

In summary, we show at the behavioral level, using a size adaptation paradigm, that sounds of different pitch (high and low frequency) can alter a visual size aftereffect. A high pitch frequency sound increases the size aftereffect while a low pitch frequency sound reduces it. A possible cortical network for this cross-modal modulation could be the visual cortex, which has been shown to be involved in purely visual size adaptation (Sperandio et al., 2012; Pooresmaeili et al., 2013; Murray et al., 2015). A possible speculation is that the same network could be involved in this audio-visual interaction. Further neurophysiological studies will be performed to validate this statement.

## Author contributions

AT and LC: Conceived and designed the project. AT: Performed experiments. AT: Analyzed data. AT, LC, and MG: Wrote and edited the manuscript. All authors gave final approval for publication.

### Conflict of interest statement

The authors declare that the research was conducted in the absence of any commercial or financial relationships that could be construed as a potential conflict of interest.
